# Electrochemical Impedance Spectroscopy (EIS): Principles, Construction, and Biosensing Applications

**DOI:** 10.3390/s21196578

**Published:** 2021-10-01

**Authors:** Hend S. Magar, Rabeay Y. A. Hassan, Ashok Mulchandani

**Affiliations:** 1 Applied Organic Chemistry Department, National Research Centre (NRC), Dokki, Giza 12622, Egypt; hendamer2000@yahoo.com (H.S.M.); ryounes@zewailcity.edu.eg (R.Y.A.H.); 2 Nanoscience Program, University of Science and Technology (UST), Zewail City of Science and Technology, 6th October City, Giza 12578, Egypt; 3 Department of Chemical and Environmental Engineering, University of California Riverside, Riverside, CA 92521, USA; 4 Center of Environmental Research and Technology, University of California Riverside, Riverside, CA 92507, USA

**Keywords:** electrochemical impedance spectroscopy (EIS), impedimetric biosensors, nanomaterials

## Abstract

Electrochemical impedance spectroscopy (EIS) is a powerful technique used for the analysis of interfacial properties related to bio-recognition events occurring at the electrode surface, such as antibody–antigen recognition, substrate–enzyme interaction, or whole cell capturing. Thus, EIS could be exploited in several important biomedical diagnosis and environmental applications. However, the EIS is one of the most complex electrochemical methods, therefore, this review introduced the basic concepts and the theoretical background of the impedimetric technique along with the state of the art of the impedimetric biosensors and the impact of nanomaterials on the EIS performance. The use of nanomaterials such as nanoparticles, nanotubes, nanowires, and nanocomposites provided catalytic activity, enhanced sensing elements immobilization, promoted faster electron transfer, and increased reliability and accuracy of the reported EIS sensors. Thus, the EIS was used for the effective quantitative and qualitative detections of pathogens, DNA, cancer-associated biomarkers, etc. Through this review article, intensive literature review is provided to highlight the impact of nanomaterials on enhancing the analytical features of impedimetric biosensors.

## 1. Overview of Electroanalytical Methods

Electroanalytical methods are considered as the most important branch of analytical chemistry, which determines characteristics along with quantity of specific analyte(s) present in an electrochemical cell. The measurement of electrochemical features taking place at the electrode interface reflects the association between the magnitude of the property measured and the concentration of particular chemical species. Compared to other analytical methods, e.g., chromatography or spectroscopy, electroanalytical techniques are much simpler and easier to miniaturize as well as being cheaper, which makes them more appropriate for rapid and accurate detection. Based on the measurable signals, electroanalytical methods are categorized as follows:

Potentiometric analysis: a reference electrode and an indicator electrode are allocated in a simple electrochemical cell whereas the difference of potential between the two electrodes is recorded to provide significant information about the sample concentration [1]. In the potentiometric technique, at zero current, the potential changes (vs. a reference electrode) are correlated to the changes of a concentration of a target analyte. The EMF of a cell depends on that concentration. Therefore, a direct calculation is easily obtained from the Nernst correlation (Equation (1)):

E_cell_ = E^0^_cell_ − (RT/nF) ln Q (1)

where E_cell_ is the measured cell potential, E^0^ is the standard cell potential, R is the universal gas constant, T is the temperature, n is the number of electron transfer, F is the Faraday constant, and Q is the reaction quotient that represents the instantaneous ratio of redox-concentrations between the anode and the cathode.

Coulometric analysis: Coulometry is a method to carry out exhaustive electrolysis of an analyte by applying constant potential onto a working electrode surface with respect to a reference electrode [2]. Coulometric titrations are common practices to measure the sample. However, the constant-potential coulometry is not subjected to the effects of interferences, since the potential of the working electrode is controlled at a value at which only a single electrochemical reaction is conducted.

Voltammetric analysis: The sample is subjected to a constant/varying potential at the electrode’s surface to record the Faradaic current produced. This technique is very important to understand the mechanisms and the kinetics of oxidation–reduction reactions and the electrochemical reactivity of an analyte [3]. The voltammetry falls into two sub-classes termed as polarography and amperometry. Polarography is a voltammetric technique in which chemical species (ions or molecules) undergo oxidation or reduction at the surface of a polarized dropping mercury electrode (DME) at an applied fixed potential vs. a reference electrode. From the resulting current–voltage (I–V) curve, both the concentration and the nature of the oxidized and/or the reduced substance(s) adsorbed at the dropping mercury electrode surface could be determined [4]. In amperometric methods, redox reactions (oxidation or reduction) of electroactive molecule(s) are measured at a constant potential. Application of voltammetry is widely exploited in biomedical diagnosis and environmental analysis [5].

Electrochemical impedance spectroscopy (EIS): EIS is one of the most important electrochemical techniques where the impedance in a circuit is measured by ohms (as resistance unit). Over the other electrochemical technique, EIS offers several advantages reliant on the fact that it is a steady-state technique, that it utilizes small signal analysis, and that it is able to probe signal relaxations over a very wide range of applied frequency, from less than 1 mHz to greater than 1 MHz, using commercially available electrochemical working stations (potentiostat). EIS theory and its data interpretation are very complicated for researchers who are not familiar with it, such as biologists, biochemists, or material scientists. Therefore, we directed our attention to explain its fundamentals in the next sections.

## 2. Basic Concept of EIS

**Figure 1:**
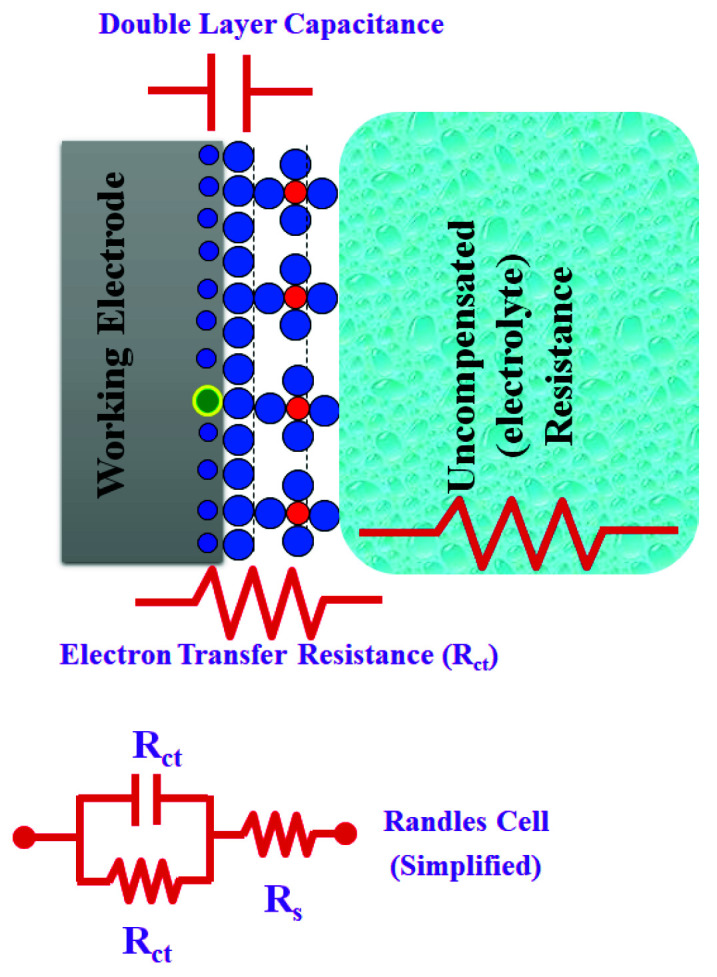
A simple scheme to descript the EIS circuit and the redox reaction takes place at the surface of working electrodes in a conventional-electrochemical cell (i.e., three-electrode system). R_ct_ is the charge transfer resistance, R_s_ is electrolyte resistance, and C_dl_ is the capacitance double layer.

In a conventional electrochemical cell, matter–(redox species)–electrode interactions include the concentration of electroactive species, charge-transfer, and mass-transfer from the bulk solution to the electrode surface in addition to the resistance of the electrolyte. Each of these features is characterized by an electrical circuit that consists of resistances, capacitors, or constant phase elements that are connected in parallel or in a series to form an equivalent circuit, as shown in Figure 1 [6]. Thus, the EIS could be used to explore mass-transfer, charge-transfer, and diffusion processes. Accordingly, the EIS has the ability to study intrinsic material properties or specific processes that could influence conductance, resistance, or capacitance of an electrochemical system. The impedance differs from the resistance, since the resistance observed in DC circuits obeys Ohm’s Law directly. A small signal excitation is applied for measuring the impedance response. The electrochemical cell response is pseudo-linear in which a phase-shift is acquired while the current response to a sinusoidal potential is a sinusoid at the applied frequency. Thus, the excitation signal is presented as a function of time, as shown in Equation (2):

E_t_ = E_0_·sin(ωt) (2)

where E_t_ is the potential at time t, E_0_ is the amplitude of the signal, and ω is the radial frequency.

The correlation between the radial frequency (ω) and the applied frequency (f) is calculated by Equation (3):

ω = 2·π·f(3)

In a linear system, the signal is shifted in phase (Φ) and has a different amplitude than I_0_ (Equation (4)).

I_t_ = I_0_ sin(ωt + Φ) (4)

**Figure 2:**
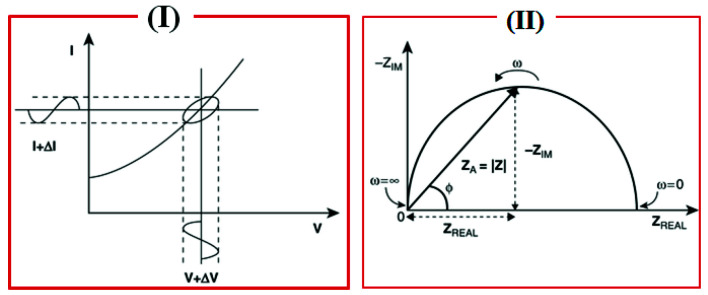
The Lissajous plot (**I**) and the Nyquist plot with impedance vector (**II**). Source: Figure modified from [8], electrochemical impedance spectroscopy (EIS) applications to sensors and diagnostics.

Thus, the impedance of the whole system can be obtained from Equation (5):

Z = E/I = Z_0_ exp(iΦ) = Z_0_ (cosΦ + isinΦ) (5)

where Z, E, I, ω, and Φ are impedance, potential, current, frequency, and phase shift between E and I, respectively. The impedance is expressed in terms of a magnitude, Z_0_, and a phase shift, Φ. If the applied sinusoidal signal is plotted on the *X*-axis and the sinusoidal response signal (I) on the *Y*-axis, the result is a “Lissajous Plot”, Figure 2(I). Before the existence of modern EIS instrumentation, Lissajous analysis was the only way for the impedance measurement [6,7].

## 3. Representations of EIS

**Figure 3:**
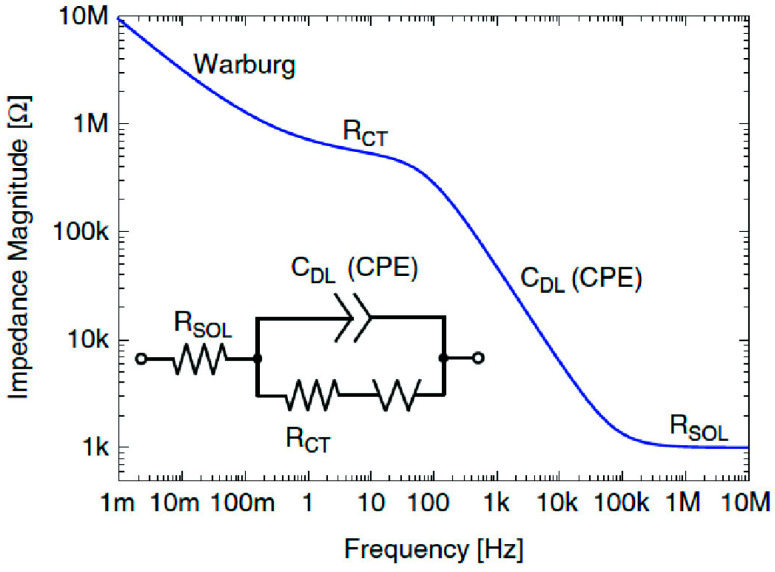
Bode plot of the impedance magnitude of the Randles equivalent model of an electrochemical interface comprising both non-Faradaic and Faradic phenomena [9].

The impedance expression is divided into a real part and an imaginary part. When the real part (Z_real_) is plotted on the *X*-axis and the imaginary part (Z_imag_) is plotted on the *Y*-axis, a “Nyquist Plot” is formed (Figure 2(II), right side). Each point on the Nyquist plot is an impedance value at a frequency point, while the Z_imag_ is negative. At the *X*-axis, impedance at the right side of the plot is conducted with low frequency, while, at the higher frequencies, their generated impedances are exerted on the left. Moreover, on a Nyquist plot, impedance can be represented as a vector (arrow) of length |Z|. The angle between this arrow and the *X*-axis is called the “phase angle”. Another way to express the impedance results is to use what is called a Bode plot, which is very common in the engineering community compared to the Nyquist plot, where the Bode plot comprises two separate logarithmic plots: magnitude vs. frequency and phase vs. frequency (Figure 3).

Practically, impedance is measured by applying a potential wave to the working electrode and recording the resulting current wave. From these two waves, Z, Φ, Z_real,_ and Z_imag_ are extracted and sketched. The spectrum is obtained by measuring these parameters for potential waves with different frequencies. The first report on electrochemical impedance spectroscopy was introduced in 1975 [10], when a small sinusoidal potential variation and the current response was measured [11,12]. In a three-electrode system, an EIS experiment is conducted by fixing an applied voltage [13]. The produced solution resistance (R_s_), charge transfer resistance (R_ct_), and Warburg impedance (W) are collected and displayed in the Nyquist plots.

There are two forms of EIS, Faradaic and non-Faradaic. In the former, impedance is produced when redox reactions take place, while the latter is a DC-based impedance, and its electrical features are produced by double layer capacitance. Electron transfer through electrode surfaces is expressed as the Faradaic current, which is exploited for quantitative analysis [14]. When frequency is plotted against phase angle, a Bode plot is configured, which is useful to find capacitance of the electrochemical systems; more information about Bode plots can be obtained from Scully and Silverman [15]. In general terms, Bode plots are used for evaluating the capacitive systems, while the Nyquist plots are typically used for analyzing the resistive processes [16]. Sum of impedances of each constituent is the overall impedance of the whole circuit that could be analyzed [8]. In that case, Ohm’s law is applied to calculate the overall impedance of a circuit with numerous components by taking the entirety of the impedances of each element:

Z_total_ = Z_1_ + Z_2_ + Z_3_ + ……………+ Z_x_ (6)

On the other hand, diffusion of molecules or redox species can create an additional resistance known as the Warburg impedance (W). This impedance is frequency dependent. Thus, at high frequencies, the Warburg impedance is small, since diffusing reactants do not have to move very far. At low frequencies, the redox molecules have the force to diffuse, thereby increasing the Warburg resistance. On the Nyquist plot, the infinite Warburg impedance displays as a tilted line with a slope of 45°. On the other hand, a phase shift of 45° is exhibited on the Bode plot referring to the Warburg effect.

## 4. EIS Equivalent Circuits

**Figure 4:**
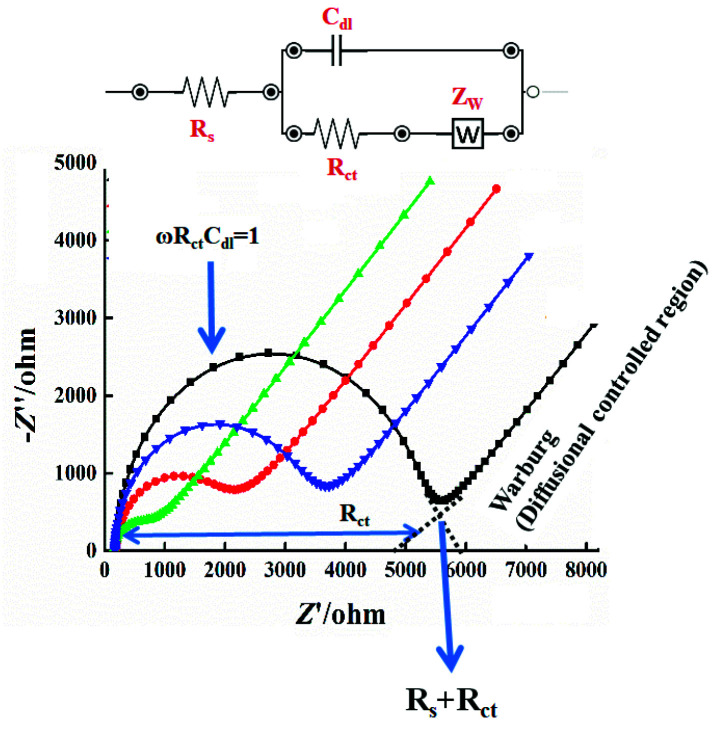
Experimental and simulated impedance spectra showing a simplified Randles equivalent circuit for an electrochemical system (original data obtained by our team). Impedance measurement is formed by Nyquist plot, which is constructed into two-dimensional X- and Y-axes for the real and the imaginary impedances, respectively. The *X*-axis is a real part of impedance (Z_real_), which represents R_s_, R_ct,_ and W values. The *Y*-axis is an imaginary part of impedance (Z_imag_). EIS experiments were conducted in a homemade conventional electrochemical cell consisting of reference, counter, and working electrodes. Ferricyanide (1 mM) was used as the standard redox probe. EIS parameters were adjusted at AC potential of 5 mV, and the applied frequency sweeps extended from 10,000 to 0.1 Hz.

Electrochemical processes associated with the electrolyte/interface and redox reactions are simulated/computed as an electric circuit (equivalent circuit) involving electrical components (resistors, capacitors, inductors). This equivalent circuit is designed and implemented to understand and evaluate the individual components of the EIS system. Resistance of solution (R_s_), double layer capacitance at the surface of the electrode (C_dI_), charge transfer resistance (R_ct_), and Warburg resistance (Z_w_) are simplified in the Randles equivalent circuits, as shown in Figure 4, [13]. Warburg resistance is the result of a diffusion process occurring at the electrode–electrolyte interface. Experimentally, the perfect capacitor does not regularly exist, thus an additional element called a constant phase element (CPE) is applied to mimic/model this non-ideal capacitance behavior. The discussed reasons behind this include surface roughness, non-homogeneity, or surface porosity of the investigated materials [17].

**Figure 5:**
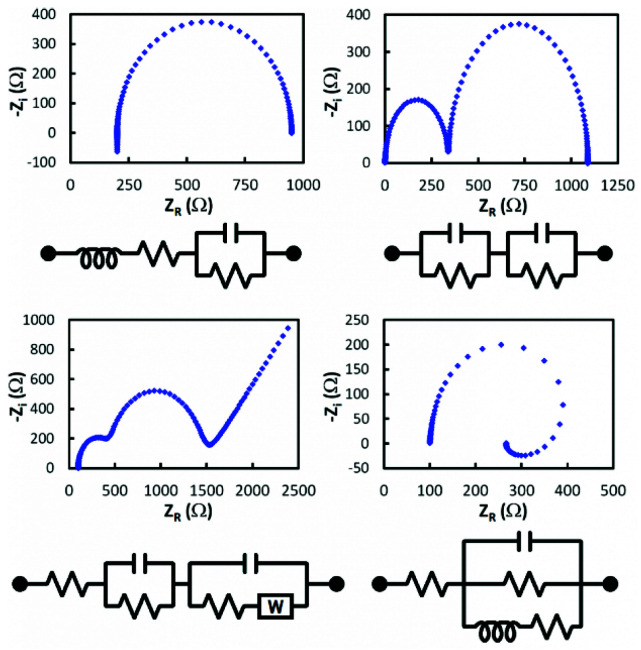
Experimental and simulated impedance spectra (examples of Nyquist plot curves and their equivalent circuits).

From Nyquist plots (practical data must be obtained first), elements of the equivalent circuit are determined and connected according to the Nyquist shape. Therefore, the EIS curve is the most important datum to be obtained first, and then surface characteristics are evaluated from fitting the electrical circuit simulation (see Figure 5). The shape of a Nyquist plot is dependent on the electrode matrix (i.e., working electrode composition) and the electrochemical responses taking place either at the surface of the working electrode or in the bulk solution. Thus, different Nyquist plot curves could be generated, e.g., a single semicircle, two semicircles, or two half-semicircles could be obtained for specific electrochemical operation [13].

Physical and chemical processes in fuel cells as well as energy storage devices can be characterized effectively using the EIS technique as a non-destructive investigating tool. Thus, the EIS can be implemented to monitor stability and performance of these materials and devices in addition to monitoring their charge transport properties [18].

## 5. Impedimetric Biosensors

Biosensors are devices used to sense the existence or the actual concentration of chemical or biological target(s). A biosensor consists of a recognition element that identifies molecular component(s) in the sample being investigated. Next, the recognition event is detected via the implementation of a diverse transducer (colorimetric, optical, electrochemical, or mass change), which collects specific signals to be processed and amplified for data interpretations. Due to small sample prerequisite, high selectivity, reproducibility, rapid detection, and high sensitivity, the biosensors become the essential diagnostic tools. Keep in mind that each target (analyte) needs certain sensing strategies and sensor configurations to be developed.

**Figure 6:**
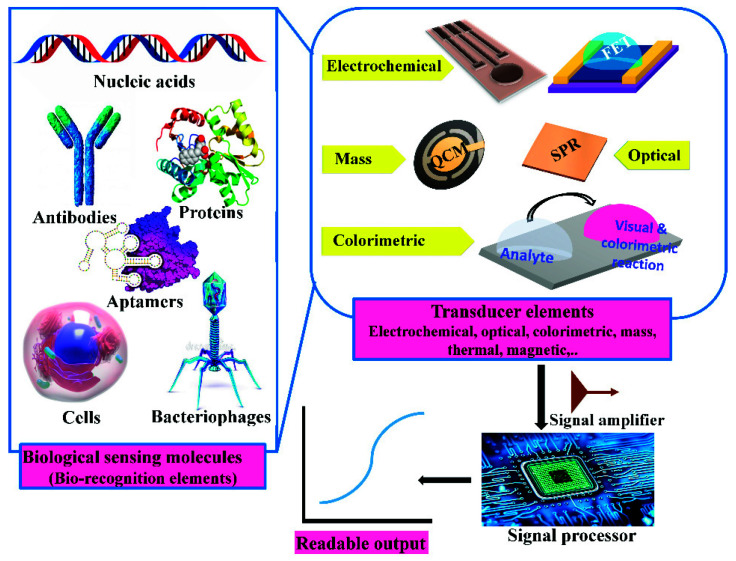
Main components of electrochemical biosensors; this figure is modified from [30].

Electrochemical biosensors (see Figure 6) have been defined as simple, easy to use, portable, cost effective, and disposable, all features that make them ideal for point-of-care devices [19]. Electrochemical sensing is made possible: a typical three-electrode electrochemical cell consists of a working, a counter (CE), and a reference electrode. In these cases, the working electrode serves as a surface on which the redox reaction takes place. Electrochemical techniques can characterize surface modifications by evaluating the electroactive area or the presence of electroactive species or by evaluating the rate of electrons exchange. Cyclic voltammetry (CV) is the most common, simple, and fast technique for acquiring qualitative and quantitative information on biological and redox reactions. The kinetics of heterogeneous electron transfer reactions, the thermodynamics of redox processes, and the coupled chemical reactions or adsorption processes can be accomplished by the CV [20,21]. The understanding of such properties could be exploited in various applications and devices such as biofuel cells or biosensors.

On the other hand, EIS is a very important method for studying and understanding the interfacial properties related to the selective bio-recognition events [22,23], e.g., the antigen–antibody capturing that occurs at the sensor surface or any other actions such as the molecular recognition of specific proteins, receptors identification, nucleic acids, or whole cells. Accordingly, several studies on EIS-based biosensors concentrated on designing aptasensors and immunosensors [24,25]. Aptamers are short single-stranded oligonucleotides (RNA or DNA) with high stability, high accessibility, and strong binding affinity [26]. Hence, aptamers are perfect for designing high performance EIS biosensors. In EIS-immunosensors, a difference in the electrical signal is created due to the kinetic binding of antibodies and its antigens at the sensors surface. As a result, electron transfer/charge transfer resistance is produced, representing the amount of bound molecules. Thus, as a label free detection, the EIS biosensors enable direct determination of biomolecular recognition actions [27]. EIS biosensors are increased significantly due to their facile manipulation, rapid response, miniaturization capability, and readiness for lab-on-a-chip integration with low cost and online measurement to detect very low concentrations [28,29].

## 6. Nanomaterials Influences the Impedimetric Biosensors

Engineering of novel electrode design using nanomaterials to functionalize the transducer surface and the tethering of receptors or the recognition elements is one of the strategies applied for the construction of electrochemical sensors. From the synthetic point of view, various techniques (physical, chemical, biological, or mixing techniques) could be used for nanomaterial synthesis. Based on the material of interest, the type of nanomaterials (e.g., 0D, 1D, 2D), the sizes, or the desired quantity, the synthesis technique is decided [31]. A wide range of nanostructured materials has been extensively used for increasing the sensor’s surface area, allowing more spaces for immobilizing the sensing element or for facilitating/amplifying the signals received from the receptor–analyte interaction [32]. Carbon based nanomaterials (e.g., fullerene (C_60_), graphene, carbon nanotubes, and carbon nanofibers) are popular for sensors surface modification due to their advantages of high electrical conductivity, large surface area, easy functionalization, and their biocompatibility.

Metal and metal oxides nanostructures are predominant materials used for electrode modification due to their electrocatalytic activity and facilitation of direct electron transfer in mediatorless biosensing systems [33,34]. Thus, ZnO, CuO, NiO, TiO_2_, and Fe_3_O_4_ were extensively used in the impedimetric biosensing to support faster electron transfer kinetics from the active sites of immobilized bioreceptors to the electrode surface, which led to synergistic enhancement in the sensing performance [35,36,37]. In one of these reports, ZnO doped-copper nanoparticles showed promising features for the development of a cost effective non-enzymatic impedimetric glucose biosensor [38].

Precious metal nanostructures including Au, Pt, Ag, or Pd were exploited for electrode modification due to their good biocompatible properties, and inertness against oxidation reactions occurred at their surfaces [39,40,41,42]. The sensors surface is functionalized by these nanostructures either directly via drop-casting or by mixing with other components (e.g., polymeric substances or sol-gel materials) in the electrode matrix [43]. Wandersonda Silva et al. developed an impedimetric sensor for tyramine based on gold nanoparticles doped-poly(8-anilino-1-naphthalene sulphonic acid) modified flat gold electrodes. The addition of gold nanoparticles increased the sensitivity of the sensor’s response with a linear range from 0.8 to 80 µM and the limit of detection of 0.04 µM [44].

In a recent study, an impedimetric sensor for total calcium detection was developed using a gold nanoparticle self-assembled monolayer to provide high sensitivity and a wide linear range from 5 × 10^−12^ to 1 × 10^−6^ mol L^−1^ with the low limit of detection of 3.6 × 10^−12^ mol L^−1^ [45].

**Figure 7:**
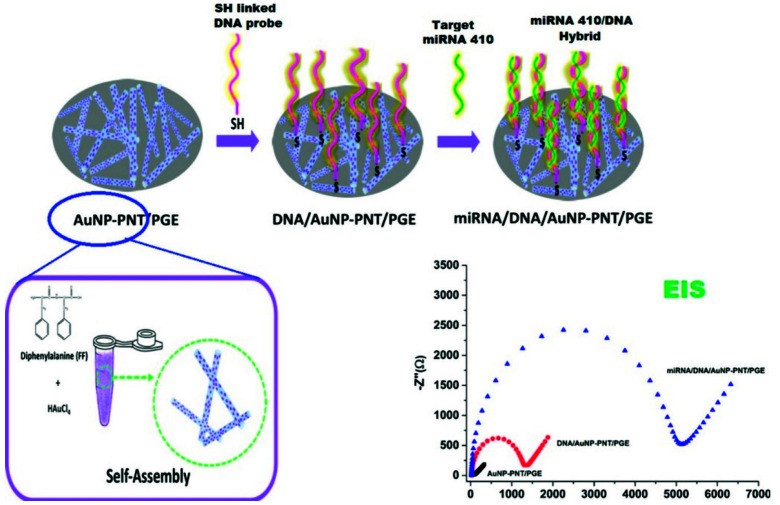
Schematic diagram of one-pot synthesized gold nanoparticle-peptide nanotube modified sensor for impedimetric recognition of miRNA, where HAuCl_4_, AuNP-PNT, and PGE represent gold(III) chloride, gold nanoparticles/peptide nanotubes, and pencil graphite electrode, respectively. Figure source: [49]. Reproduction of the figure was granted from the publisher; the reference number is 210801-024789.

**Figure 8:**
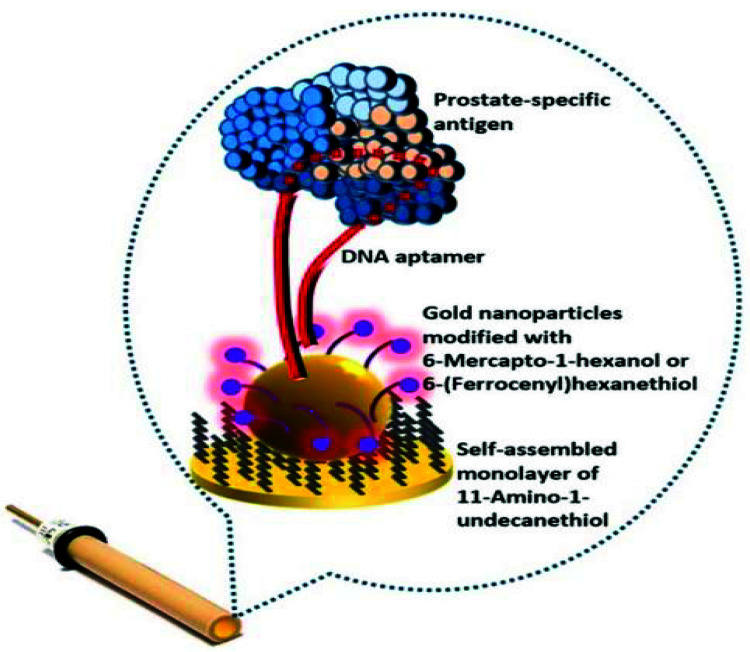
Schematic diagram of self-assembled gold nanoparticles for impedimetric recognition and detection of a prostate cancer biomarker. Figure source: [50]. Reproduction of the figure was granted from the publisher; the reference number is 210801-024863.

On the other hand, a labeled electrochemical system for the detection of DNA breast cancer using AuNPs was established. The concept is built on the fact that electrons from the redox mediator (Fe(CN)_6_) are transferred to the electrode through the ssDNA conjugated AuNPs [46]. By applying this approach, breast cancer gene *BRCA1* was detected without any signal amplification. Consequently, Gao et al. developed a DNA sensor in which the AuNPs were displaced by target DNA, and it was utilized for the mediated impedimetric detection with a very high sensitivity, whereas the detection limit was 50 fM [47]. These DNA sensors consisted of simple structural designs of the capture probes with minimum steps of preparation, which are great advantages for sensor fabrication. However, in these sensors, the DNA–gold binding is attained through the interaction between gold and bases of DNA via the electrostatic interaction, and, hence, the success of binding between the AuNPs and the DNA is necessary for the sensor fabrication where the size and the charge on the AuNPs become significant. Accordingly, functionalized AuNPs were prepared to solve the above issues and achieved successful binding between AuNPs and DNA. Other classes of nanostructured electrodes were fabricated using a hybrid of metal and metal oxides to enhance the electron communication rate between redox active species and electrode surface. In this regard, magnetite and gold nanoparticles (Fe_3_O_4_/Au) modified electrodes were implemented for quantification of DNA of the hepatitis B virus [48]. The Fe_3_O_4_/Au modified electrode accelerated the charge transport and increased the sensitivity for DNA hybridization. Other studies attempted to couple different materials to maximize electron transfer. On the other hand, gold nanoparticles-assembled peptide nanotubes modified with graphite electrodes were introduced for the impedimetric analysis of circulating miRNA-410 secreted by prostate cancer cells (Figure 7). The modified electrode showed high sensitivity and low detection limit to be applicable in the impedimetric recognition of the target miRNA [49]. In another report, a prostate specific antigen as a biomarker for prostate cancer was detected using the aptasensor modified with gold nanoparticles (Figure 8) [50]. The anti-PSA DNA aptamer was exploited for both square wave voltammetry (SWV) and impedimetric detections. Using the Au-NPs, a significant improvement in the limit of detection was obtained.

Further, detection of the HIV-1 gene using a label-free DNA impedimetric sensor was assisted by the AuNPs/carbonized glass fiber-coal tar pitch-electrodes [51]. This sensor offered a limit of detection of 13 fM. The thiol-modified electrodes were prepared using amine-crosslinking chemistry, and the coated surfaces with AuNPs self-assembled were highly conductive.

Besides, bacterial impedimetric biosensors for fast detection of major foodborne pathogens (*E. coli* O157:H7) were made using the immunoglobulin G (IgG) antibody. A well-defined order of self-assembled layers of thiolated protein G (PrG)@ gold nanoparticles modified electrodes was exploited for the IgG immobilization. The AuNPs-based biosensor exhibited a very high selectivity towards the target pathogen over other bacteria such as *Staphylococcus aureus* and *Salmonella typhimurium.* Moreover, the sensor provided a limit of detection of 48 colony forming units (cfu mL^−1^), which is three times lower than that of the planar gold electrode biosensor (140 cfu mL^−1^). Therefore, the improved impedimetric performance was attributed to the synergistic effect of the AuNPs-PrG-thiol framework [52]. Furthermore, AuNPs-protein G was exploited for building up a sensitive EIS biosensor for the detection of cancer biomarker epidermal growth factor receptors. The biosensor was tested on different samples obtained from human plasma and brain tissue, which encouraged it to be applied in clinical screenings and prognoses of tumors [53]. Arginine-functionalized gold nanoparticles for the detection of DHEAS, a biomarker of pediatric adrenocortical carcinoma, was developed, and the EIS was applied as the measuring technique [54]. This immunosensor was developed using anti-DHEA IgM antibodies as the bio-recognition element immobilized at the glassy carbon electrode functionalized with AuNPs. A linear relationship between ΔR_ct_ and DHEAS concentration was verified in the range from 10 to 110 μg/dL, with a LOD of 7.4 μg/dL. Besides the good sensitivity, the immunosensor displayed accuracy, stability, and specificity to detect the DHEAS in real patient plasma samples.

As a sensing strategy, from the above mentioned finding, the use of nanomaterials for developing EIS biosensors did not only increase the electrode surface but also allowed rapid and sensitive detection of desired analytes.

## 7. Carbon-Based Impedimetric Biosensors

As a result of the advances made in developing electrochemical biosensors, carbon nanomaterials have continuously expanded in various aspects, from raw electrode materials to surface modifications at the nanoscale. Graphene and carbon nanotubes are the most common carbon materials used for constructing EIS biosensors due to their high electrochemical activity, high electrical conductivity, large surface area, ease for functionalization, and biocompatibility [55,56]. Thus, derivatives of carbon materials including graphene oxide (GO) and reduced graphene oxide (rGO) have been utilized in electrochemical sensing. By exfoliation of graphite in water using sonication, a single layer to a few layers (nano-sheets) of graphene oxides could be produced [57]. The GO is strongly affected by the density of oxygen-containing groups because of the higher negative charge of the graphitic surface that causes a higher charge transfer resistance [58]. Therefore, the formed GO has less electrical conductivity than the reduced GO. On the other hand, the hydrophilicity of graphene oxides is increased due to the presence of oxygen-containing functional groups on its surface, which provides high dispersion and more surface area for molecular binding [59].

Those oxygen-containing groups could be reduced electrochemically by applying a suitable electrical potential to create a reduced graphene oxide (rGO) with excellent conductivity [60]. Alternatively, thermal, chemical, or combined chemical and thermal methods could be used to achieve the complete reduction and exfoliation of graphene oxide [61,62,63]. Composition and functionalization of graphene-related nanomaterials have a strong effect on the immobilization of biorecognition elements. Generally, EIS measurements are performed in Faradaic mode using electrochemical redox probes (electron mediators) to focus on the R_ct_ variations between the solution and the electrode interface [64]. Using graphene quantum dots and gold-embedded polyaniline nanowires, impedimetric sensors for the hepatitis E virus (HEV) were designed. HEV virus particles were captured by the immobilized antibody to provide high sensitivity. The sensor linearity response in serum samples ranged from 10 fg mL^−1^ to 100 pg mL^−1^. Ultimately, the proposed sensor was suggested as a robust probe for rapid HEV detection [65].

Three-to-four-layers of reduced graphene oxide were fabricated and used as a sensing platform for hairpin DNA [66]. The detection of complementary ssDNA was more robust and sensitive with LOD of 6.6 pM, while the single rGO-layer platform gave LOD of 50 nM. Moreover, a DNA-based rGO sensor was developed by Hu’s group. In this approach, positively charged moieties were introduced for the chemical coupling of DNA probes [67]. Additionally, rGO-nanoparticles were formed on indium tin oxide (ITO) flat electrodes using cyclic voltammetry. This sensor platform was applied for the direct impedimetric detection of C-reactive protein (CRP) in human serum samples with a detection limit of 0.08 ng mL^−1^ [68].

Another label-free impedimetric biosensor was constructed for the detection of low-density lipoprotein (lipid or LDL) cholesterol. Anti-lipoprotein B-100 was covalently immobilized on amine-functionalized reduced graphene oxide using EDC/NHS coupling chemistry to show a high sensitivity with the limit of detection of 5 mg/dL of LDL molecules within 250 s [69].

Referring to the other carbon-based materials, single-walled carbon nanotubes (SWCNTs) and multi-walled carbon nanotube (MWCNTs) are two basic forms of the carbon nanotubes (CNTs) which possess almost all of the aforementioned advantages of graphene materials, making them the second most popular nanomaterials for electrochemical biosensors. Different electrochemical properties could be induced upon changing the orientation and the arrangement of CNTs on the electrode surface [69]. CNTs can be utilized either as a nanocarrier due to the large surface area and the easy amendment or as an electrochemical nanoprobe-based sensor. Growth of Au nanoparticles onto the vertically aligned MWCNTs was reported to detect a specific TP53 gene sequence [70]. Hence, the EIS was used to evaluate the DNA hybridization events related to the TP53 gene, and it exhibited outstanding response towards the target TP53 mutation. The detection limit was 10 nM, and the sensitivity enhancement was due to the synergistic interactions of the aligned MWCNTs arrays with the well-distributed AuNPs. On the other hand, gold-coated SWCNTs as a microelectrode were exploited to detect complementary 10-base DNA whereas the charge transfer resistance of the sensor was varied with respect to the target DNA concentrations. The synergistic interactions of horizontal SWCNT arrays with the AuNPs were the reason behind the major enhancement occurring in the sensitivity of this sensor. By using this methodology, the sensor gave a detection limit of 100 nM for single base mismatch DNA. As the authors claimed in their report, each gold-coated SWCNT acted as a separate micro-electrode, which could be used to detect fewer than six DNA molecules in a 1 mL sample [71]. On the other side, heavy metal (Pb^2+^) was detected indirectly based on its inhibition effects on choline oxidase using the MWCNTs conjugated with AuNPs [72]. A unique nanocomposite made of Au NPs/MWCNTs-graphene quantum dots was produced by Ghanavati et al. [73] for the label-free detection of a prostate specific antigen (PSA) in clinical samples with a limit of detection of 0.48 pg/mL. Based on the mentioned EIS applications of the carbon-based materials (graphene and carbon nanotubes or their nanocomposites), they share interesting features in common, including thermal properties and them being electronic and excellent mechanics. However, graphene affords more opportunities in biosensing applications, as it can be greatly produced at a low cost in large-scale construction.

## 8. Nanowires-Based Impedimetric Biosensors

Among the nanomaterials used, nanowires (NWs) have emerged as a new class of promising functional nanomaterials [74,75]. Certain aspects render considerable interest in the use of NWs as electrochemical transducers. Those unique NWs characteristics are unidirectional conduction channels, diameters and dimensions appropriate to the size and the shape of target molecules, in addition to the outstanding electrical transport property. Both conducting and semiconducting nanowires were reported, including gold nanowires for Alzheimer’s disease detection [76], gallium nitride nanowires for nucleic acid detection [77], titanium oxide nanowires for bacterial sensing, and silicon nanowires for the detection of hepatitis B and liver cancer biomarkers (α-fetoprotein (AFP)) [78]. NWs EIS-based sensors have different constructions and configurations that affect their applications. One-dimensional (1D) nanostructure wires are used for semiconducting field effect devices, while 3D collections of nanowires are implemented as sensing ensembles (nanowires array) [75]. A nanowire array performance is highly dependent on the fabrication techniques that control the structural parameters, such as diameter, length, ordered orientation, and crystallinity structure. For example, a DNA biosensor based on vertically aligned gold nanowires array by electrodeposition was developed by Ramulu et al. [77]. From Au-NWs morphological studies, the nanowires were strongly attached to the flat gold surface and well-aligned, which provided more electron transfer ability to detect the specific hybridized DNA in a low concentration. In another study, different metal nanowire types with different lengths were grown on paper substrates using electrodeposition template-assisted and simple adhesive tape-based patterning at room temperature. The approach exhibited excellent electrode tissue impedance suitable for recording electrocardiogram signals without any wet-gel adhesives [79]. Moreover, tellurium doped zinc oxide (Te-ZnO NWs) nanowires were used for highly sensitive impedimetric DNA sensors in a label-free approach for hepatitis B virus (HBV) detection [80]. The HBV-DNA sensor responded to the complementary target in a concentration range from 1 pM to 1 μM, with the detection limit of 0.1 pM.

Additionally, functionalized ZnO nanorods and carboxylated graphene nano-flakes were used as a composite deposited on an indium tin oxide (ITO) substrate for the covalent immobilization of *E. coli* O157:H7-specific DNA probe. The obtained impedimetric results displayed linear response in a wide range of DNA concentrations (10^−16^ M to 10^−6^ M) with a detection limit of 0.1 fM.

Another NWs-based impedimetric platform was constructed for the detection of a cardiac biomarker (Troponin-I (cTnI)) using tungsten trioxide nanowires (WO_3_-NWs). A layer of 3-aminopropyltriethoxy saline was deposited onto the WO_3_ surface. The impedimetric response demonstrated high sensitivity with the linear detection range of 0.01–10 ng/mL. The sensor’s surface modification with the WO_3_-NWs is a very promising platform for the development of a point-of-care biosensing device for cardiac detection [81]. With a controlled thermal synthesis of WO_3_, a thin layer of 3-aminopropyltriethoxy saline (APTES)-functionalized WO_3_ was placed on an ITO substrate, while the covalent binding of a cTnI antibody onto a functionalized surface was carried out using EDC-NHS chemistry. The impedimetric response of this immunosensor was suggested as a promising platform for cardiac detection. It is worth mentioning here that the cardiac troponins (cTnI) are considered as the gold standard biomarkers for myocardial injury [82,83].

## 9. Nanocomposite-Based Impedimetric Biosensors

Nanocomposites are materials (two or more phases) with different types or different structures engineered in nanoscale dimensions [21,84]. Use of nanocomposites (nanoparticles [85], nanosheets [86], or nanotubes [87]) is one of the most trending strategies in the development of impedimetric biosensors due to their unique electrochemical, mechanical, thermal, optical, and catalytic features.

Fusco et al. developed an impedimetric sensor for the detection of a tumor associated antigen expressed in malignant cells by electrochemical deposition of a polyaniline/graphene oxide nanocomposite on indium tin oxide (PANI/GO@ITO) [88]. An increase in the amplitude of the impedance signal was obtained resulting from the over-expression of the target cancer biomarker (CSPG4) in both cell culture medium and cell-lysate protein. This biosensor was recommended to be an alternative to ELISA and flow cytometry. In another study, AuNPs/PANI nanocomposite was used as a non-enzymatic EIS glucose sensor, which give a linear range from 0.3–10 mM and lower detection limit of 0.1 mM [89]. Using this nanocomposite, enzymatic-less glucose detections were enabled, and the use of glucose oxidase was avoided. In another report, a thin film of a PANI-Ag-Cu nanocomposite was deposited on glass substrates using spin coating technique and was applied in the impedimetric detection of *E. coli* [90]. Furthermore, electropolymerization of a poly-(aniline-co-3-aminobenzoic acid) (PANABA/AuNPs) nanocomposite material was conducted for the immunodetection of 2,4-dichlorophenoxy acetic acid herbicide in spiked samples [91]. The established impedimetric immunosensor showed a limit of detection of 0.3 ppb, which is lower than herbicide emission limits. In conclusion to this section, nanocomposite-based EIS sensors are very promising but need continuous efforts to develop novel materials for various target detection.

For another clinical diagnosis, the level of vitamin D deficiency was tracked in blood samples using Au nanoparticles functionalized with a nanocomposite consisting of a hybrid of graphitic carbon nitride (GCN) and β-cyclodextrin (β-CD). This label-free impedimetric immunosensor provided high sensitivity signals with the LOD of 0.01 ng/mL [23].

## 10. Nanopores and Nanochannels Array

Conceptually, formation of nanopores and nanochannels arrays on the electrode matrix or surfaces could be accomplished to create nanoelectrode arrays with exceptional ion transfer and mass transport properties. This could be exploited for designing high performance electrochemical sensors [92,93,94]. In nanochannel-based biosensing systems, the concentration of analytes is quantified by measurement of electrical conductance change between two separated conductive compartments, where such analytes penetrate/diffuse to be firmly anchored in the nanochannels. Applications of the nanochannel platforms in biosensing are diverse, ranging from DNA [95], cancer biomarkers [96], enzyme [97], and pathogens [98] to gases and vapors of small molecules such as polychlorinated biphenyls [99]. In nanochannel-based biosensing systems, the impedance sensing can be performed by Faradaic or non-Faradaic models [100]. To avoid the use of redox species, the non-Faradaic models are more common. Thus, they are well-suited for the detection of binding events inside the nanopores. Anodic aluminum oxide nano-porous membranes are the most popular nanochannel-based platforms used with EIS technique [101]. The preparation of anodic aluminum oxide nano-porous membranes was performed by using electrochemical anodization, and they present an attractive method to develop nanopore biosensing devices due to their uniform pore size, high surface area, high aspect ratio, and inexpensive preparation [102].

Nagaraj and his group successfully improved a nanochannel sensor for pharmaceutical contaminants detection in water. Ibuprofen concentration was measured in water samples with the LOD of 0.25 pg mL^−1^ [100]. Furthermore, different pathogens (such as *S. aureus* and *E. coli* O157:H7) were detected, where selectivity and sensitivity reached a detection capability of 10^2^ CFU/mL [103].

For adjusting the nanostructures to be suitable for non-Faradaic sensing models, Kant et al. studied the impact of pore dimension on the performance of biosensing by attaching streptavidin and biotin covalently with a selected monoclonal antibody of the targeted organisms [103]. The anodic aluminum oxide nano-porous membrane was prepared with different pore sizes and lengths followed by the functionalization of streptavidin on the inner surface of the pores, creating a covalent binding site for biotin molecules. As a recommendation, lowering nanochannel diameters (less than 10 μm) is not favorable for non-Faradaic EIS detection due to the high resistance and the long time required for analyte diffusion inside the channels. Hence, optimization of nanochannel dimensions is a critical factor that has significant influence on the performance of nanopore-based electrochemical biosensing devices. Nanochannel-based biosensing approaches are a very promising research area with tremendous potential applications. In conclusion to this section, the construction of nanodevices enabling real-time detection systems for sensing of selected target(s) occurring at confined spaces or interfaces is a significant challenge. Thus, the nanochannel-based electrochemical sensors could be constructed and optimized.

## 11. Nanogap Electrodes

As a suggested solution to eliminate the negative effect of the double layer, the debye length was increased as the ionic strength of the solution was increased. However, this required additional separation and purification steps for medium replacement. Alternatively, decreasing the electrode separation distance permitted the electric field uniformity within the target medium. This concept inspired researchers to construct nanogap electrodes [104]. A nanogap-based biosensing platform represents the organization of two conductive electrodes separated by a distance of no more than 300 nm. Nanogab sensors were applied in the detection of chemical or biological interactions taking place at the interface. The distance limit of 300 nm was defined, since it characterizes the upper limit of the electrical double layer, which formed at all charged conductive sides in aqueous media [105,106].

By exploiting this advance approach, DNA [107], protein [108], and other biological molecules [109] were detected. The 1D nanogap with point-type gap junctions is classically intended for single molecule detection by applying AC potential to produce resistive quantities. The 2D nanogap with band-type gap junctions and the 3D nanogap with surface-type gap junctions are intended for monitoring of biological parameters (e.g., binding efficiency of biomolecules) from complex impedance response via AC measurement techniques [110]. To overlap the double layer effect as well as to reduce the ohmic drop between electrodes, the gap size was defined to be less than 100 nm. Graphene nanogap electrodes (GNEs) were used for the detection of streptavidin–biotin biomolecular interactions. The electrodes showed high-affinity interactions of streptavidin–gold nanoparticles to the biotin-functionalized nanogaps. This platform is recommended as a biosensor for the detection of other affinity-based biomolecular interactions, such as nucleic acid, antigen–antibody, or chemo-selective interactions [111]. In another study, nanogap interdigitated electrode (IDE) arrays with assisted gold nanoparticles were used to enhance the sensitivity of detection [85].

Nanogap biosensors were able to detect specific proteins in serum or blood directly, and they were used also in early disease detection. Despite nanogap biosensors development, there is still no commercial device available in the research phase to fulfill the selectivity and the reproducibility issues due to the technical limitations in mass fabrication. However, the perspective of a small volume, highly sensitive, label-free, low power consumption and all-electrical biosensing device is still appealing. Ultimately, the nanogap electrodes are very important tools for the investigation of material properties at the nanometer scale or at the molecular level. Therefore, they might be considered as building blocks for the construction of nano-circuits and nanodevices.

## 12. Conclusions, Remarks and Future Perspective

A biosensor is a self-contained integrated device based on a biological recognition element(s) (e.g., enzymes, nucleic acids, antibodies bacteria, lectins, cells) to provide precise quantitative or semi-quantitative analytical information. Among the most common electrochemical biosensors, impedimetric biosensors have attracted a great deal of attention. Accordingly, they have been widely exploited to detect enzymatic activity, DNA hybridization, antibody–antigen recognition, and binding affinity. From our sorted information in this review, nanomaterials such as metals, metal oxides, carbon, nanowires, nanocomposite, nanopores, nanochannels array, and nanogap species have been used for developing EIS biosensors. The use of such nanomaterials provided several improvements in terms of analytical features, including enlarging sensor surface area, increasing sensitivity and selectivity, amplifying the electrochemical signals, and increasing rapidity of the sensor’s response. Table 1 provides a summary of all the reported materials in this review. Dealing directly with samples on the chip, the EIS could be provided as a portable device for instantaneous and simple point-of-care (POC) in hospitals, airports, and hotspots.

## Figures and Tables

**Table 1:** A summary of the reported materials for target analysis using the EIS.

Electrode Material	Target of Analyte	Detection Limit	Linear Range	Ref
GCN-β-CD/Au nanocomposite	Vitamin D deficiency detection	0.01 ng/mL	0.1 ng/mL to 500 ng/mL	[23]
Reduced graphene oxide and gold nanoparticles	Detection of penicillin G	0.8 fM	1.0 fM to 10 μM	[26]
Reduced graphene oxide (RGO) with iron oxide nanoflowers (IONFs)	Removal of the synthetic organic dye reactive blue			[36]
Copper-doped Zinc oxide nanoparticles (Cu-ZO)	Detection of glucose	10^−9^ M	10^−9^ M to 10^−5^ M	[38]
Silver nanoparticles	DNA sensor			[39]
Platinum nanomaterials	Listeria detection		1 × 10^−1^ M to 1 × 10^−4^ M	[40]
Microwires formed by platinum nanoparticles	Detection of acetamiprid and atrazine	1 pM	10 pM to 100 nM	[41]
Aluminum oxide (AAO) gold nanoparticles (GNPs)	Detection of genomic length hepatitis B virus (HBV) DNA	10^2^ copies/mL	10^2^–10^3^ and 10^3^–10^5^. copies/mL	[43]
Gold nanoparticle-poly-(8-anilino-1-napthalene sulphonic acid), AuNP-PANSA	Determination of tyramine (Tyr)	0.04 µM	0.8 to 80 µM	[44]
Gold Nanoparticles	Calcium detection	3.6 × 10^−12^ mol L^−1^	5 × 10^−12^–1 × 10^−6^ mol L^−1^	[45]
Gold nanoparticles (GNPs)	Label-free DNA detection	1 pM breast cancer gene BRCA1		[46]
Gold nanoparticles (AuNPs)	DNA detection		50 fM to 1 pM	[47]
Gold nanoparticle	Hepatitis B virus DNA		8.3 (±0.1)×10^−13^ to 6.4 (±0.2)×10^−7^ M	[48]
Gold nanoparticle assembled peptide nanotube (AuNP-PNT)	miRNA 410	3.90 fM	10 fM to 300 pM	[49]
Gold nanoparticles	Detection of a prostate cancer biomarker	10 pg/mL	10 pg/mL to 10 ng/mL	[50]
Gold nanoparticles (AuNPs)	Detection of HIV-1 DNA	13 fM	0.1 pM and 10 nM	[51]
Gold nanoparticles (AuNPs)	Detection of *E. coli* O157:H7	48 cfu mL^−1^	up to 10^7^ cfu mL^−1^	[52]
Gold nanoparticles	Cancer marker epidermal growth factor receptor in human plasma and brain tissue		1 pg mL^−1^–1 μg mL^−1^	[53]
Arginine-functionalized gold nanoparticles (AuNPs-ARG)	Detection of DHEAS, a biomarker of pediatric adrenocortical carcinoma	7.4 µg dL^−1^	10.0 to 110.0 µg dL^−1^	[54]
Graphene quantum dots and gold nanoparticle-embedded polyaniline nanowires	White spot syndrome virus	48.4 DNA copies/mL.	1.45 × 10^2^ to 1.45 × 10^5^ DNA copies/m	[62]
Reduced graphene oxide-nanoparticle (rGO-NP)	Detection of C-reactive protein	0.06 and 0.08 ng mL^−1^	1 ng mL^−1^ and 1000 ng mL^−1^	[68]
Reduced graphene oxide	Detection low-density lipoprotein (LDL) molecules	5 mg/dL		[69]
Gold nanoparticles/aligned carbon nanotubes	Detection of cancer, TP53 gene mutation	1.0 × 10^−17^ M	1.0 × 10^−15^-1.0 × 10^−7^ M	[71]
Multiwalled carbon nanotubes (MWCNT) and gold nanoparticles (GNP).	Choline determination	0.6 μM	3 to 120 µM	[72]
Au nanoparticles/MWCNTs- graphene quantum dots nanocomposite	Detection of prostate specific antigen	0.48 pg/mL	1–10000 pg/mL	[73]
Pd Nanowires	H_2_-based electrochemical biosensor	0.04 ng mL^–1^	0.1–50 ng mL^–1^	[74]
Diamond nanowires decorated with nickel nanoparticles	Detection of immunoglobulin G (IgG)	0.3 ng mL^−1^ (2 nM)	300 ng mL^−1^ (2 μM)	[75]
Reduced graphene oxide and gold nanowires	Detection of Alzheimer’s diseasequantification of serum microRNA-137	1.7 fM	5.0 to 750.0 fM	[76]
Gold nanowires array electrode (AuNWsA)	Enhanced electrochemical detection of nucleic acid	6.78 × 10^−9^ M		[77]
Silicon-on-isolator-nanowires (SOI-NWs)	Detection of the hepatitis B marker HBsAg	up to 10^−14^ and 10^−15^ M for HBsAg and AFP, respectively		[78]
Tellurium doped ZnO nanowires	Hepatitis B virus DNA detection	0.1 pM	1 pM to 1 μM	[80]
WO_3_ nanorods	Detection of a cardiac biomarker		0.01–10 ng/mL	[81]
Polyaniline/graphene nanocomposite	Detection of chondroitin sulphate proteoglycan 4			[88]
Gold nanoparticles/polyaniline	Glucose detection	0.1 mM	0.3 to 10 mM	[89]
PANI-Ag-Cu nanocomposite	Detection of *E. coli*			[90]
AuNPs-functionalized PANABA-MWCNTs nanocomposite	2,4-dichlorophenoxy acetic acid detection	0.3 ppb		[91]
Alumina nanopore	DNA	2.5 nM		[95]
Gold nanoparticles	Direct detection of a cancer biomarker in blood	52 U mL^−1^ of CA15-3		[96]
Nanoporous membrane with hyaluronic acid (HA)	Detection of pathogenic bacteria in whole milk	10 cfu/mL	10–10^5^ cfu/mL	[98]

## Data Availability

Not applicable.
